# Design of a binary programmable transmitarray based on phase change material for beam steering applications in D-band

**DOI:** 10.1038/s41598-024-53150-9

**Published:** 2024-02-05

**Authors:** Samara Gharbieh, Jorick Milbrandt, Bruno Reig, Denis Mercier, Marjolaine Allain, Antonio Clemente

**Affiliations:** Univ. Grenoble Alpes, CEA, Leti, 38000 Grenoble, France

**Keywords:** Engineering, Materials science, Physics

## Abstract

This paper introduces the design of a reconfigurable transmitarray operating within the D-band frequency range (110–170 GHz). The transmitarray unit cell is composed of three metal layers and two quartz dielectric substrates. It achieves a 1-bit phase shift resolution through the alternating states of two innovative switches integrated into the active transmitting patch of the unit cell. To address the challenge of miniaturization in the D-band, compact switches compatible with the proposed unit cell dimensions are introduced. These switches are constructed using phase change materials (PCM) that change between amorphous and crystalline states when exposed to heat. The paper includes a full-wave simulation of the unit cell, demonstrating an insertion loss below 1.5 dB across a wide frequency band of 27%. Additionally, a 10 $\times$ 10 elements transmitarray is synthesized using a numerical tool and its theoretical results are compared to full-wave electromagnetic simulations for validation purposes. The results indicate that by incorporating the proposed switches into the unit cell, the transmitarray achieves promising reconfiguration capabilities within the D-band. Moreover, the paper presents the architecture of a command line designed to bias the PCM switches. Notably, this command line represents a novel approach, as it enables individual biasing of each PCM switch using direct current (DC). The influence of these command lines on the transmitarray’s performance is thoroughly investigated. Although there is a compromise in the 1-dB gain bandwidth, the overall behavior of the transmitarray remains encouraging.

## Introduction

Manipulating and shaping electromagnetic (EM) fields at Terahertz (THz) frequencies represents a key technique utilized for the analysis and detection of various biological and chemical components. THz wavelengths facilitate EM interactions with the target, showcasing a wide band behavior that is essential in the development of high-resolution and high-performance wireless systems. Hence, THz frequency band is used in several applications across diverse fields such as spectroscopy, astronomy, space observation, remote sensing, pharmaceutical and industrial control, imaging, holography, and radar/sensing^1–5^. Recently, the THz band has gained significant attention in next-generation wireless communications^6–8^, mainly due to its wide bandwidth capabilities, extending up to hundreds of GHz. These bandwidths surpass the conventional millimeter-wave spectrum, which typically operates within a range limited to tenths of GHz. Nonetheless, notable challenges such as considerable free-space path loss, dispersion, and molecular absorption limit the effective use of THz waves, making them particularly suitable for ultra-high-throughput communications within short-range indoor environments^9,10^.

Programmable EM surfaces present a revolutionary technology capable of actively altering and controlling electromagnetic fields. Recently, EM surfaces and RIS (reconfigurable intelligent surfaces) have gained significant attention in telecommunications and signal processing research^11^. Especially, EM surface technology stands as a promising candidate for future communication networks due to its potential to enhance wireless localization precision^12^ and create a controllable, and smart EM environment^13^. Such a paradigm for a disruptive communication network of the future could employ an extremely large number of RIS and would require a drastic reduction of the power consumption (by more than two orders of magnitude) to be an attractive and widely adopted solution.

Typically, an EM surface operates as a reflective, transmissive, or transflective programmable surface, employing radiating elements or unit cells with a period ranging from one-tenth to one-half of a wavelength^14^. When the unit cell measures less than a quarter of a wavelength, it is termed a metasurface, meta-screen, or meta-lens^15–17^. Otherwise, it’s known as a reflectarray (RA) or transmitarray (TA), depending on its operational principle and feeding mechanism. Additionally, leaky-wave EM apertures fall under the category of RIS^18^. In contrast to classical phased array antennas^19–21^ using local phase shifters and power amplifiers for controlling phase and amplitude distribution on the radiating surface, RIS manipulate EM characteristics (e.g., impedance, current distributions) of unit cells via locally programmed RF switches, variable capacitive loads, or tunable materials^14^. Unlike phased arrays, EM surfaces demonstrate high efficiency, eliminating the need for complex and lossy feed networks. Consequently, EM surfaces serve as efficient, energy-conserving platforms for holographic beam shaping, exhibiting wide band, high-gain characteristics, and programmable features in the THz band.

For EM surfaces designed to function in the microwave and millimeter wave spectrum, conventional practices involve heterogeneous integration of tunable devices^22,23^. This approach includes discrete electronic devices (e.g., varactors^24–29^, RF MEMS switches^30,31^, p-i-n diodes^32–49^) flip-chipped onto the unit cell, typically constructed using printed circuit board (PCB) technologies. Additionally, tunable substrates like liquid crystals^18^ or ferroelectrics^50^ could be used in such designs. In the THz regime, implementing heterogeneous integration using packaged semiconductors presents significant challenges due to limited unit cell dimensions and the demand for high efficiency, low insertion and integration losses. Addressing this limitation involves exploring technologies based on various tunable materials categorized by their activation mechanism: electrical, mechanical, optical, and thermal/chemical^51,52^.

Among the existing approaches, two of them are the most suitable. The first involves non-volatile/bi-stable materials like phase change^53–59^ and 2D materials^60–64^, although with limited practical demonstrations. While PCMs exhibit promise for energy-efficient EM surfaces due to their non-volatile nature, they face limitations such as scalability issues and the inability for individual control over each unit cell^55,56^. In the case of 2D materials, a graphene substrate can modify the resonance frequency of radiating elements; however, this approach, similar to varactor-based EM surfaces, the achieved bandwidth is less than 10%^60^. The second technology involves silicon-based processes like CMOS^53,65,66^, known for scalability and real-time control advantages. Nonetheless, the EM properties of silicon-based materials constrain radiating surface performance, significantly reducing bandwidth due to high relative permittivity (> 10). Furthermore, the material conductivity ($\gamma = 6 e^-14 Sm^{-1}$) introduces insertion losses (e.g., over 2 dB on the reflection/transmission coefficient of a meta-element at 0.3 THz^66^).

In order to overcome the mentioned challenges, we utilize PCM-based switches to design a reconfigurable TA operating in the D-band within this study. GeTe (Germanium Telluride) is specifically chosen for its stability and advantages at high frequencies. While the GeTe switch technology was initially developed in a prior work^67^, this study presents an optimized design to meet D-band unit cell requirements. Notably, excluding our preliminary conference paper^68^, this represents the first TA design employing PCM switches that do not act as conductor metals in their amorphous state, differentiating it from previous approaches^69,70^. These switches are locally integrated into each unit cell to ensure a 180$^{\circ }$ relative phase shift. The unit cell consists of three metal layers separated by dielectrics: the receiving patch, located on the feeder side, connected by a metallic via hole to the transmitting patch containing the integrated switches , and the ground plane positioned within the middle layer. Moreover, a novel command line design is introduced for the first time in the literature, allowing individual biasing of each switch without relying on optical stimuli, as typically associated with PCMs^69^. The updated unit cell, incorporating command lines, is simulated in the full-wave EM simulator and compared with the initial unit cell without command lines. Although insertion losses increased by 1.2 dB and the $-1$ dB gain decreased by 10%, the 180$^{\circ }$ phase shift remains unaffected. Subsequently, the TA constructed using the updated unit cell is simulated and compared with the one built using the initial unit cell. While the maximum gain remains at 15.5 dBi at 140 GHz, the $-1$ dB bandwidth experiences a reduction. However, these trade-offs are acceptable in pursuit of developing a practical solution.

The paper starts by introducing the operational concept of the binary programmable unit cell, showing the innovative PCM switch design and displaying the unit cell simulation results. Subsequently, it goes into the TA model, its design, and its performance characteristics. The final section covers the design of the command lines and their impact on the TA performance. In conclusion, the paper discusses the outcomes of this research and offers insights into future directions.

## Results

### Design of the binary programmable unit cell

**Figure 1:**
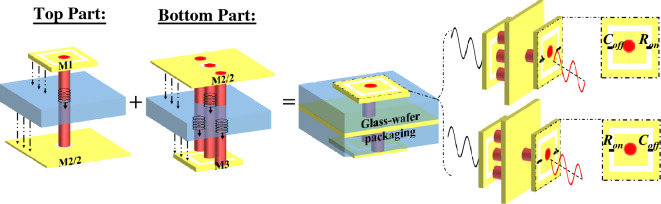
A binary programmable transmitarray unit cell architecture.

As previously explained, transmitarray (TA) antennas gained popularity due to their capability to implement relatively simple and low-loss reconfigurability functions, providing real-time beam scanning, beamforming, and polarization control. In the implementation procedure of a TA, the first step is to design the unit cell. In this case, the unit cell shall be designed such that it provides a phase shift through electronic tuning. In other words, the unit cell architecture should provide a local switching capability as illustrated in Fig. 1. When the switch is “ON”, it is equivalent to a resistor denoted by $R_{\text{on}}$. Whereas when the switch is “OFF”, it is equivalent to a capacitor denoted by $C_{\text{off}}$. In order for the switch to be useful in the programmable unit cell design, it should present an acceptable $R_{\text{on}} \cdot C_{\text{off}}$ product. Moreover, the unit cell must generate a discrete number of states given by $2^n$, e.g., two phase states (0$^{\circ }$, 180$^{\circ }$), or four phase states (0$^{\circ }$, 90$^{\circ }$, 180$^{\circ }$, and 270$^{\circ }$), where $n$ denotes the number of bits of the programmable unit cell.

#### Novel PCM switch and unit cell operating principle

In order to enable switching capabilities for the unit cell in the D band, several challenges need to be overcome. The traditional switches used in the literature in the design of reconfigurable TA such as varactors, MEMS, and p-in diodes, have large sizes compared to the size of the unit cell in the D band, which is proportional to the wavelength.

**Figure 2:**
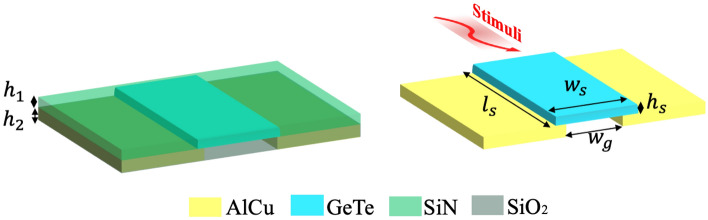
The GeTe based switch stack-up and design.

In our work, in order to overcome the aforementioned challenge, a PCM based RF switch was designed. The switch is made of GeTe due to its low figure of merit in terms of $R_{\text{on}} \cdot C_{\text{off}}$ product ($\approx$10 $fF\Omega$ (femto Farad-Ohms)). The advantages of having a low $R_{\text{on}} \cdot C_{\text{off}}$ are numerous. It primarily results in reduced insertion losses, minimized power consumption, and enhanced radiation efficiency. This improvement is attributed to the lower $R_{\text{on}}$ value, resulting in minimal dissipation of power. Additionally, lower $R_{\text{on}} \cdot C_{\text{off}}$ products facilitate efficient reconfiguration of the TA, enabling precise phase control. This precision ensures more accurate beam steering and beam shaping. The GeTe switch is activated with a thermal stimuli which could be applied through an electrical or an optical pulse. The GeTe electrical properties can then change in order to switch from an amorphous state (GeTe OFF) to a crystalline state (GeTe ON) or vice versa. This phenomenon is depicted in Fig. 2. To enable the transition from amorphous to crystalline state, the GeTe switch is heated at a temperature $T_1$ for a duration $d_1$ (100 *ns* to 1$\mu$s). Note that this temperature $T_1$ should be lower than the fusion temperature and higher than the crystallization one ($\approx$ $300\,^\circ$C). On the other hand, to change the state from crystalline to amorphous, the switch is heated for a duration $d_2$ of 100 *ns* at a temperature $T_2$ higher than the fusion temperature ($\approx$ $750\,^\circ$C).

The switch stack-up and design are shown in Fig. 2. The GeTe material (with dielectric constant $\epsilon _r = 36$) is placed on the top of two metal (aluminium and copper alloy AlCu) arms of thickness $h_2$, separated by a distance $w_g$. The metal is embedded in a silicon dioxide ($SiO_2$: dielectric constant $\epsilon _r = 4$) layer of the same thickness $h_2$. The GeTe switch is also embedded in a silicon nitrate ($SiN$: dielectric constant $\epsilon _r = 7$) layer of thickness $h_1$. The switch is of cubic form with dimensions $l_s \times w_s \times h_s$. All the geometrical parameters are summarized in Table 1.

**Table 1:** The geometrical parameters of the switch are expressed in mm.

$h_1$	$h_2$	$l_s$	$w_s$	$w_g$	$h_s$
$4\times 10^{-4}$	$6\times 10^{-4}$	$2\times 10^{-2}$	$3\times 10^{-3}$	$1\times 10^{-3}$	$3\times 10^{-4}$

The dimensions of the GeTe switch are set in order to achieve the required $(C_{\text{off}}, R_{\text{on}})$ values. Namely, when the GeTe is in its OFF state, it is equivalent to a capacitor $C_{\text{off}}$ of $10 \text{fF}$ whereas when it is in its ON state it is equivalent to a resistor $R_{\text{on}}$ of $1 \Omega$. It is noteworthy to mention that the alternation of the GeTe states between OFF and ON (amorphous and crystalline) has a significant effect on the resistivity of the switch. This is provoked due to the change of the material resistivity from $1.85\times 10^{5}~S/m$ in the crystalline state to $1.85~S/m$ in the amorphous state.

**Figure 3:**
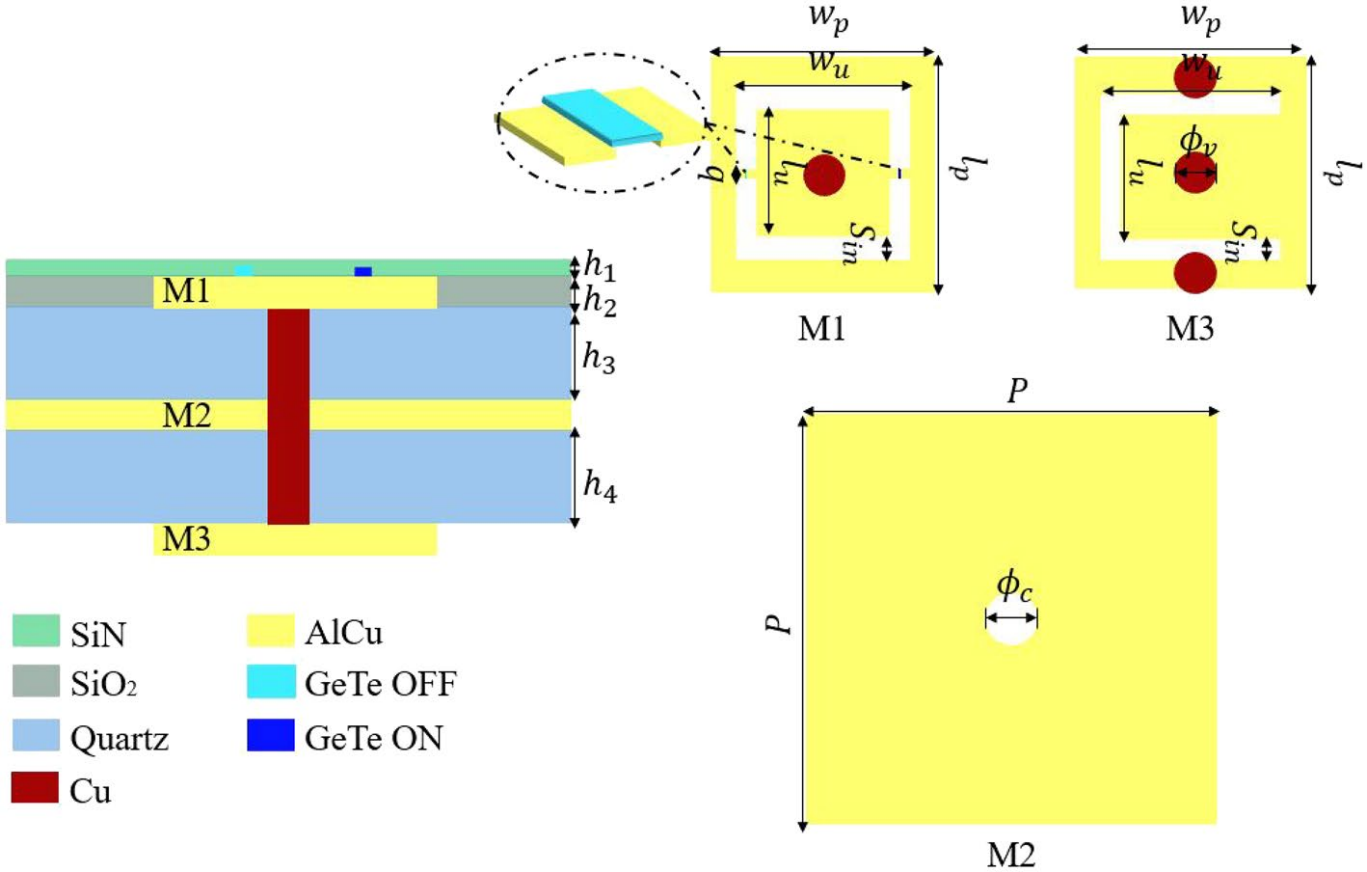
The unit cell stack-up and detailed structure.

The proposed TA unit cell falls into the AFA (Antenna-Filter-Antenna) concept. Thus, the unit cell will have at least three metal layers of the same thickness $h_2$: (1) the receiving patch (Rx), placed on the feeder side, which is a passive patch; (2) the ground plane, which will be in the middle layer; and (3) the transmitting patch (Tx), placed in the free space side, which is an active patch with the PCM switches. The structure is a glass-wafer topology, hence the metal layers will be separated by two quartz glass dielectrics, with the following electrical properties : $\epsilon _r = 3.8$ and $\tan \delta = 0.0001$. In Fig. 1, the glass-wafer packing steps are presented. First, the TGV (Through Glass Vias) are fabricated and filled with metal in the glass substrate. Then, the second step is to prepare the AlCu metal layers M1 and M3, i.e., the Tx and Rx patches (see Table 2). The Tx (M1) and Rx (M3) patches will be each packaged with a layer of the ground plane of thickness $h_2/2$ (denoted by M2/2 in Fig. 1). In the end, the two halves are combined, to obtain the complete stack-up of the unit cell. The detailed structure of the unit cell is illustrated in Fig. 3. The cell is optimized in the D-Band (110–170 GHz), hence the periodicity is set to $P=\lambda _0/2$ (1.07 mm) at the central frequency of 140 GHz.

**Table 2:** The geometrical parameters of the unit cell structure expressed in mm.

$h_1$	$4\times 10^{-4}$	$l_u$	$0.22$
$h_2$	$6\times 10^{-4}$	$w_u$	$0.32$
$h_3$	$0.2$	$b$	$0.03$
$h_4$	$0.2$	$S_{in}$	$0.05$
$l_p$	$0.44$	$\phi _v$	$0.08$
$w_p$	$0.44$	$\phi _c$	$0.1$

In Fig. 3, the architecture of each metal layer is depicted. The active Tx layer (M1) consists of a rectangular patch of dimension $w_p \times l_p$ loaded by an O-slot. It is connected by a metallic via hole of diameter $\phi _v$ to the Rx patch (M3), which is a rectangular patch of dimension $w_p \times l_p$ loaded by a U-slot. The central via passes through the ground plane in the middle layer, and in order to avoid the short circuit of the via a contour hole is perforated in the ground plane of diameter $\phi _c$. The Rx layer is connected to the ground plane with two via holes of diameter $\phi _v$ for biasing purposes. The detailed geometrical parameters of the cell are summarized in Table 2.

To make the unit cell electronically programmable, two GeTe switches that were developed specifically for the unit cell design (shown in Fig. 2) were integrated in the Tx patch. This configuration of the GeTe switches allows generating a binary state. While alternating the ON (crystalline) and the OFF (amorphous) states of the GeTe switches, the O-slot of the Tx patch will be transformed into a U-slot either in the direction of the one in the Rx patch or in the opposite one. This configuration generates a symmetrical behavior, where the scattering parameters of the two states have equal magnitudes and a 180$^{\circ }$ degrees phase difference.

#### Unit cell simulation and performance

**Figure 4:**
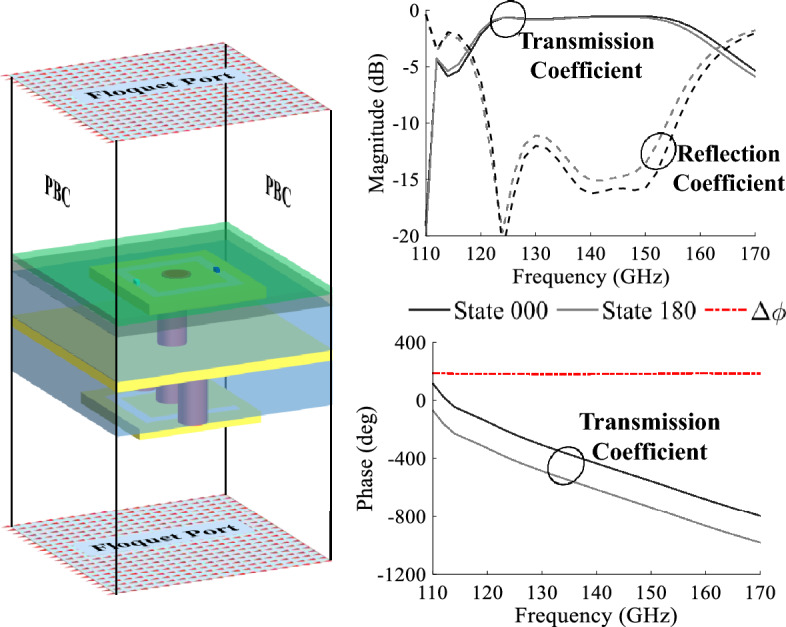
The unit cell simulation setup in HFSS, the magnitudes of the transmission and reflection coefficients and the phase of the transmission coefficients.

The proposed unit cell has been simulated in the full wave simulator Ansys Electronics—HFSS. The main purpose of the simulation is to demonstrate the capabilities of the unit cell with GeTe switches to produce the needed phase shift, which is created by alternating the switch states. Thus, the unit cell will create two phase states called: “State 000” equivalent to the combination where the GeTe OFF is on the left and the GeTe ON is on the right of the O-slot, and the “State 180” is the alternate case. The simulation setup is sketched in Fig. 4. Two Floquet ports are defined on the top and bottom sides of the unit cell with periodic boundary conditions (PBC). Both State 000 and State 180 were simulated in the same presented setup over the frequency band between 110 and 170 GHz.

**Figure 5:**
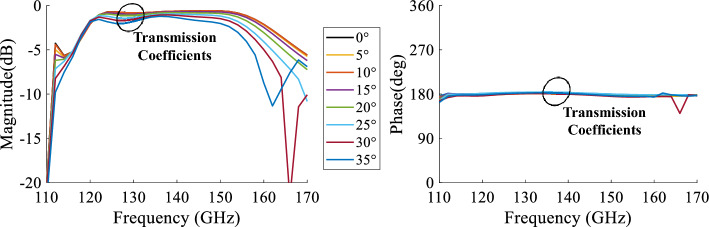
The magnitudes and the phase shift ($\Delta \phi$) of the transmission coefficients of the unit cell simulated under several incidences.

The unit cell in State 000 and State 180 is simulated separately under normal incidence. In Fig. 4, the magnitudes of the transmission coefficient and reflection coefficient are shown for both states. This figure shows that the cell exhibits very low insertion loss, 0.69 dB at 140 GHz. The insertion losses are lower than 1.5 dB over a 27% bandwidth for both states. In Fig. 4 as well, the phases of the transmission coefficient for the two unit cell states and the phase difference $\Delta \phi$ are shown. It is seen from the plots that the unit cell can produce two phase states with low insertion losses and a phase difference around 180$^{\circ }$ over a relatively large frequency range. In order to further investigate the unit cell behavior, the incidence angle is varied between 0$^{\circ }$ and 35$^{\circ }$ under the same simulation setup. The simulation results of the transmission coefficient magnitudes and phase shifts $\Delta \phi$ as a function of the angles of incidence are presented in Fig. 5. The unit cell presents an S21 magnitude lower than $-2$ dB for a wide frequency band of 30 GHz (120–150 GHz; i.e, 22.22%) for an incidence angle up to 35$^{\circ }$. At the central frequency 140 GHz, the maximum insertion losses for an incidence of 35$^{\circ }$ are of value 1.4 dB. The important fact is that the phase shift $\Delta \phi$ remains constant with value 180$^{\circ }$ over the frequency band independent of the incidence angle. These results demonstrate the robustness of the unit cell behavior in terms of low insertion losses and stable phase shift regardless of the incidence angle.

### Binary phase gradient flat lens design procedure

The unit cell performances show that the proposed configuration can be a great fit for the TA design, since it has low insertion losses over a wide frequency band and a constant phase shift ($\Delta \phi$) of 180$^{\circ }$. In order to synthesize and optimize a TA, a hybrid full-wave methodology based on both the analytical expression and full wave simulation was previously developed among our group. This model was validated with measurement results up to 300 GHz in^71–73^. Thus, the unit cell full-wave simulations data were used to generate the TA using the aforementioned tool.

#### Transmitarray model and synthesis

**Figure 6:**
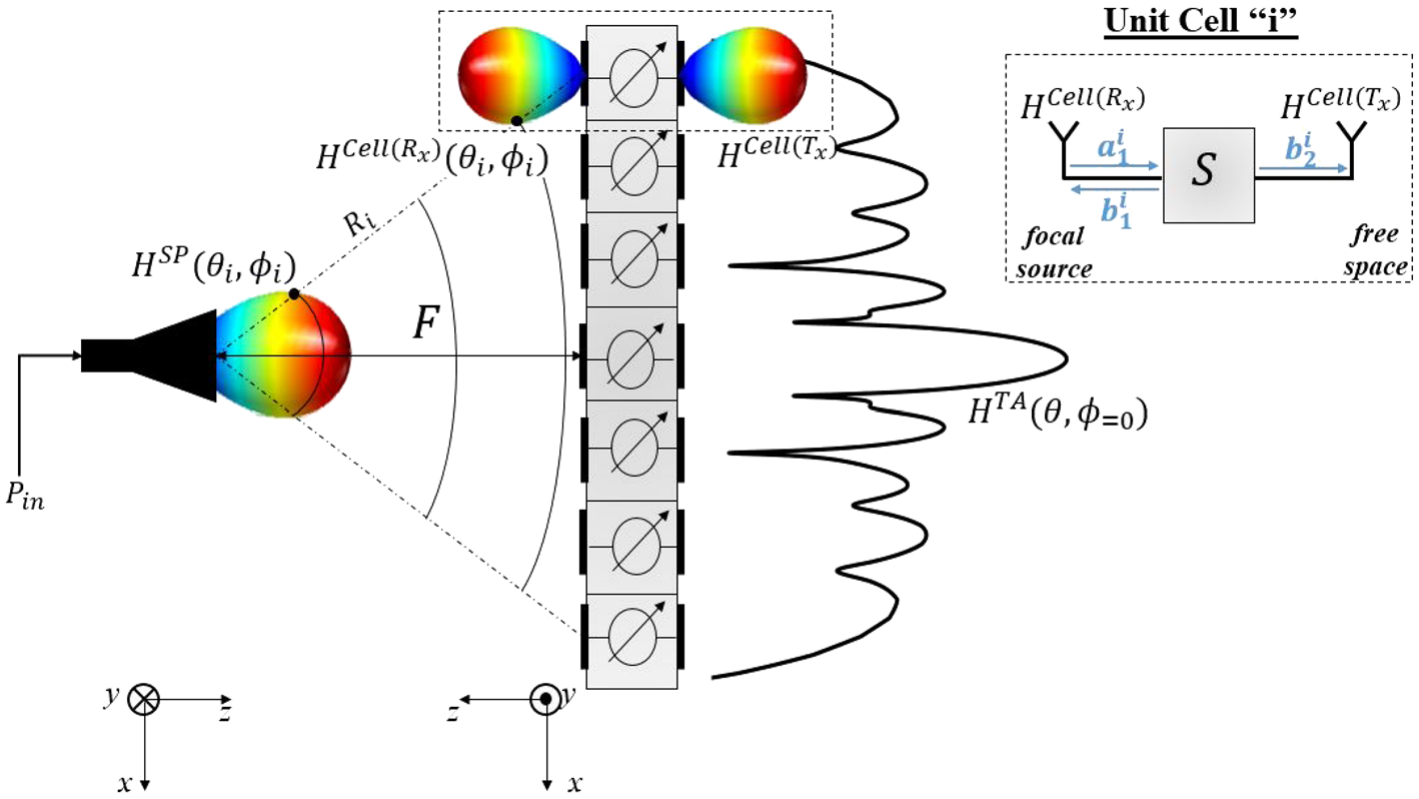
The full scenario of the transmitarray considered in the analytical calculation in the Matlab model.

The TA scenario is represented in Fig. 6. A focal source, which is a pyramidal horn antenna placed at a focal distance $F$, illuminates a quasi periodic lens (the TA). This lens consists of $M \times M$ unit cells, where each unit cell follows one of the two binary states (States 000 or 180). The developed tool requires the simulated complex E-field of the focal source and the unit cells as input. The tool then outputs the adequate phase gradient on the TA aperture, the TA radiation pattern, and other TA performance indicators (gain bandwidth, efficiency, SLL, etc.).

The focal source is simulated in the full-wave simulator Ansys Electronics HFSS, and its radiation patterns are exported. The radiation pattern of the focal source in the direction of the $i$th unit cell of the TA is denoted by $H^{SP}(\theta _i,\phi _i)$ in Fig. 6, where $(\theta _i,\phi _i)$ corresponds to the direction of the unit cell with respect to the focal source. The input power on the horn antenna is denoted by $P_{in}$.

**Figure 7:**
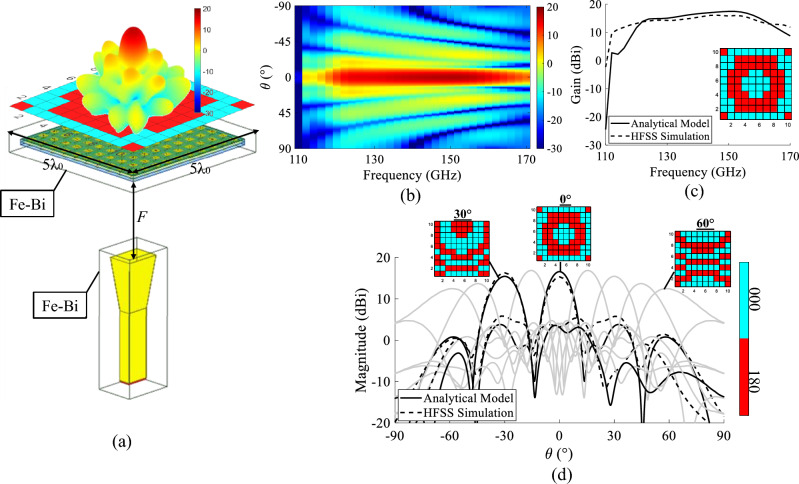
(**a**) The transmitarray simulation setup in HFSS. (**b**) The gain in cut-plane $\phi =0$ in terms of $\theta$ and the frequency calculated in Matlab. (**c**) The comparison of gain variation in the frequency band for the broadside transmission in the cut-plane $\phi =0$ between HFSS and Matlab. (**d**) The comparison of the radiation pattern for several transmissions between HFSS and Matlab.

The unit cell is composed of two antennas connected through a 2-port network (Antenna-Filter-Antenna) presented on the right top of Fig. 6. The port network contains the $S$-parameters, exported from the full-wave simulation under normal incidence whose setup is presented in Fig. 4. Since the study of the unit cell showed that its performance is not affected by the incidences, the $S$-parameters under normal incidence are only considered.

Since the actual incident power captured by each unit cell is dependent of the radiation pattern of the unit cell on the Rx side, additional simulations have been done to export the complex radiation patterns on both the Rx and Tx sides. The complex radiation pattern of the $i\textrm{th}$ unit cell in the direction of the focal source is denoted by $H^{Cell(R_x)}(\theta _i,\phi _i)$. Consequently, the exported values of $H^{Cell(R_x)}(\theta _i,\phi _i)$ take into account the effect of the adjacent cells (periodicity $P$) and the reduction of the effective area. Therefore, the incident wave $a_1^i$ received by the $i$th unit cell, with $i=1,2,\ldots ,M^2$, is given by

1 $$\begin{aligned} a_1^i=\sqrt{P_{in}}~.~ \frac{\lambda e^{-j2\pi R_i/\lambda }}{4\pi R_i}~ .~ H^{SP}(\theta _i,\phi _i)~.~H^{Cell(R_x)}(\theta _i,\phi _i), \end{aligned}$$

where $\lambda$ is the wavelength and $R_i$ is the distance between the focal source and the center of the $i$th unit cell.

The calculation of the incident wave $a^i_1$ and its phase at each unit cell center will generate the phase distribution on the top of the lens. For each unit cell on the lens (TA), the state (State 000 or 180) is selected in order to compensate the phase distribution created from the incident wave and to fit best the required transmission direction, i.e., transmission to broadside or steered beam to a certain direction. Since the designed unit cell provides two phase states (State 000 and 180), a binary phase quantization is considered to approximate the phase distribution. The quantization $Q$ is given by

2 $$\begin{aligned} Q(\angle S^i_{21}) = \left\{ \begin{array}{@{}cl@{}} 0, &{} \text{if } -\frac{\pi }{2} \le \angle S^i_{21} < \frac{\pi }{2},\\ 180, &{} \text{otherwise}. \end{array}\right. \end{aligned}$$

where $\angle S^i_{21}$ is the required phase shift for the $i$th unit cell of the TA. The reflected and transmitted complex waves $b_1^i$ and $b_2^i$ of each unit cell (illustrated in Fig. 6) are computed as follows,

3a $$\begin{aligned} b_1^i= S_{11}^i a_1^i, \end{aligned}$$

3b $$\begin{aligned} b_2^i= S_{21}^i a_1^i. \end{aligned}$$

Finally, the radiation pattern of the TA, denoted by $H^{TA}(\theta ,\phi )$, is calculated by summing the contribution of each unit cell. This is done by considering the transmitted wave $b_2^i$ of each unit cell and its radiation pattern on the free space side $H^{Cell(T_x)}$ (see Fig. 6) following the arrangement of unit cells. Therefore, the complex radiation pattern of the TA for a given pair $(\theta _j,\phi _j)$, with $-\frac{\pi }{2}\le \theta \le \frac{\pi }{2}$ and $0\le \phi \le \pi$, is given by

4 $$\begin{aligned} H^{TA}(\theta _j,\phi _j)= \sum _{i=1}^{M^2} b_2^i. H^{Cell(T_x)}(\theta ,\phi ) \end{aligned}$$

The Matlab tool can ensure a full analytical study and optimization of the TA. Several parameters can be studied such as the optimal focal distance $F$. The far-field results could be calculated once the phase distributions are generated for any steering angle.

#### Electromagnetic validation

A transmitarray of $$10 \times 10$$ elements ($$5\lambda \times 5\lambda $$ , $$\lambda $$ at 140 GHz) is synthesized using the MATLAB tool discussed in the previous section. The size of the TA is then equal to $$D=$$10.7 mm. The focal distance is optimized to 8mm such that $$F/D = $$0.8. In Fig. 7, the gain radiation patterns on the horizontal plane for all the steering angles between $$-60^{\circ }$$ and 60$$^{\circ }$$ with a step of 15$$^{\circ }$$ are shown, in addition to the frequency response of the TA at broadside. The colormap of the 2D plot of the gain in terms of frequency (GHz) and $$\theta $$ $$^{\circ }$$ in Fig. 7b (calculated in the case of the broadside transmission) shows that the TA is able to transmit the beam towards $$\theta = 0$$ $$^{\circ }$$ for all the frequency band with a gain of 16.6 dBi at 140 GHz.

The full TA is simulated using the Ansys Electronic HFSS for two different phase distributions. The first phase distribution corresponds to a broadside transmission and the second one to a transmission towards 30$^{\circ }$. The setup of the full-wave simulations for both 0$^{\circ }$ and 30$^{\circ }$ is shown in Fig. 7a. The feeder is a pyramidal horn antenna with a nominal gain of 10 dBi placed at the focal distance $F$= 8 mm. The cell distribution (cell State 000 is represented in a blue square where as the cell State 180 is a red square) is applied for both cases respecting the two phase distributions calculated for 0$^{\circ }$ and 30$^{\circ }$ transmissions. The TA and the horn antenna are placed inside two hybrid boxes of Finite Element Boundary-Integral equation solution (Fe-Bi). Instead of considering one large radiation box, two smaller hybrid boxes are used to enable applying the finite element method around the TA and horn only. This setup is adopted in order to reduce the time required for simulation. The TA size is set to $10 \times 10$ elements in order to validate the MATLAB tool results, and therefore has an acceptable size for full-wave simulation.

The HFSS simulation results are then compared to the MATLAB theoretical results. Figure 7d presents the patterns at the central frequency 140 GHz calculated using the Matlab tool for transmission angles between $-60^{\circ }$  and  +60°. The patterns for the two transmission angles 0$^{\circ }$ and 30$^{\circ }$ exported from the HFSS simulations are also reported. The plots exported from HFSS (dotted plots) show good agreement with the plots (lined plots) calculated in MATLAB and the pointing angle in both cases is correct. The gain versus the frequency in the case of the broadside transmission is shown in Fig. 7c. The gain at 140 GHz exported from HFSS is of 15.8 dBi (equivalent to an aperture efficiency (The aperture efficiency of the TA is defined as the ratio between the maximum gain and the maximum directivity of a uniform amplitude aperture with the same physical dimensions as the TA.) of 12.0%), which is slightly lower than the gain calculated in MATLAB (16.6 dBi; equivalent to an aperture efficiency (The aperture efficiency of the TA is defined as the ratio between the maximum gain and the maximum directivity of a uniform amplitude aperture with the same physical dimensions as the TA.) of 14.5%). The $-1$ dB gain bandwith is of 12.8% and the gain versus frequency calculated in MATLAB is in good agreement with the results exported from HFSS.

**Figure 8:**
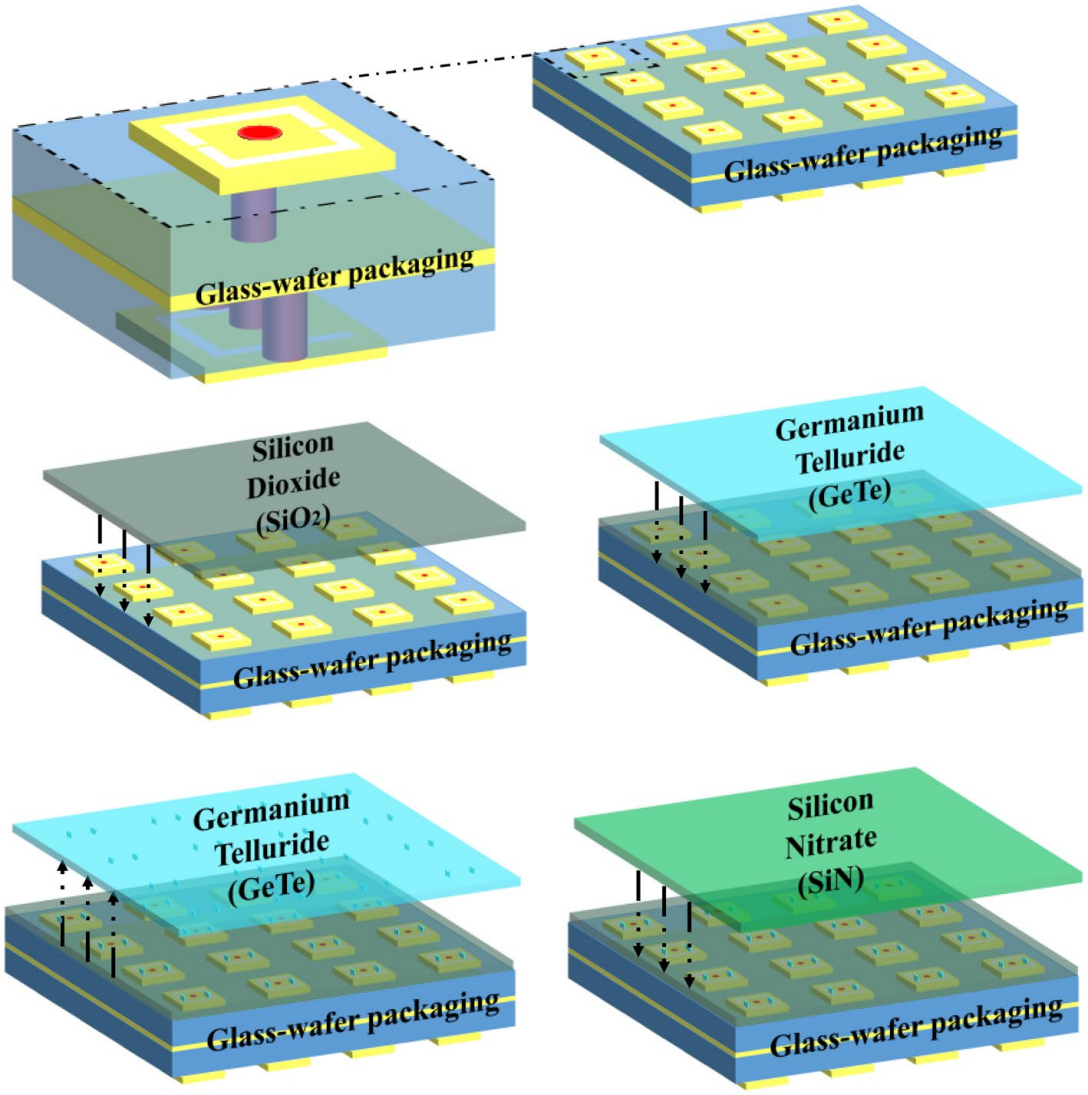
The steps of the fabrication procedure.

### Impact of bias lines and potential fabrication technique

The simulations results shown in the previous section demonstrate the feasibility of designing a reconfigurable TA using the developed PCM-based unit cell. We expect the fabrication technique to follow from a series of steps. The wafer-like stack-up of the unit cell is made and then the layers of the switches are added. The patches, ground plane and dielectrics are packed according to the stack-up explained in Fig. 1. Afterwards, the layer of silicon dioxide ($SiO_2$ is added to soak the top metal layer (Tx). Then, the GeTe is added as shown in Fig. 8. To localize the GeTe as two switches (rectangle of $l_s \times w_s \times h_s$) on each unit cell, the unused GeTe material is removed and only the switches remain. Finally, the silicon nitrate ($SiN$) is added.

**Figure 9:**
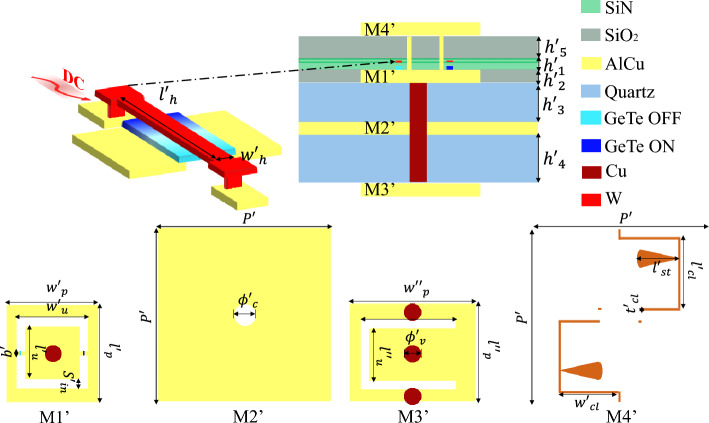
The stack-up and structure of the updated unit cell including the command lines.

**Table 3:** The geometrical parameters of the unit cell with command line structure in mm.

$h'_1$	$6\times 10^{-4}$	$\phi '_c$	$0.16$	$b'$	$0.03$	$l'_{cl}$	$0.45$
$h'_2$	$6\times 10^{-4}$	$\phi '_v$	$0.08$	$S'_in$	$0.03$	$l'_{st}$	$0.25$
$h'_3$	$0.15$	$w'_p$	$0.41$	$w''_p$	$0.6$	$t'_{cl}$	$5\times 10^{-3}$
$h'_4$	$0.2$	$w'_u$	$0.27$	$w''_u$	$0.4$	$w'_{cl}$	$0.25$
$h'_5$	$3\times 10^{-3}$	$l'_p$	$0.4$	$l''_p$	$0.42$		
$P'$	$1.07$	$l'_u$	$0.27$	$l''_u$	$0.2$		

**Figure 10:**
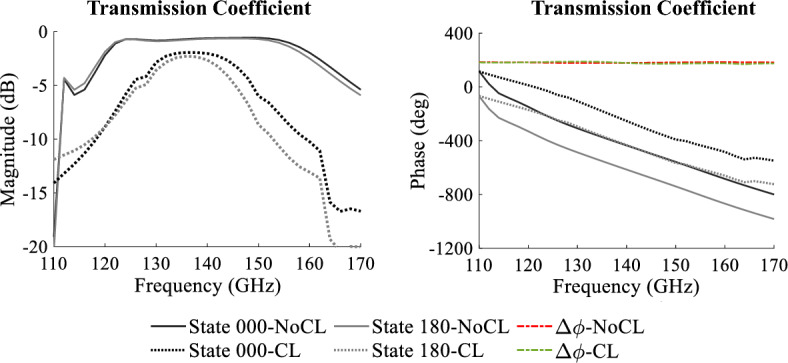
The comparison of the transmission coefficients magnitudes and phase for the unit cell without command lines (NoCL) and the unit cell with command lines (CL).

The study holds promise for more in-depth exploration, particularly regarding the activation process. The GeTe activation in the literature is often made by the application of laser stimuli^69^. In the following, we propose an updated version of the unit cell structure in order to activate the novel GeTe switches separately by the application of a thermal stimuli through a heater. To this end, a command line design is proposed to induce a direct current into the heater. The details of the new version of the unit cell structure and the effect of the command line on the unit cell are discussed in the subsequent sections.

#### Command line effect on the unit cell performances

As previously mentioned, the goal is to have an independent control of each switch in order to alternate the GeTe states between (OFF/ON) and create the needed state of each unit cell. A stimuli is needed in order for the GeTe to change from amorphous to crystalline. In this work, in order to heat the GeTe, a heater of tungsten ($W$) material is added on top of each GeTe switch, as seen in Fig. 9. A thin layer of silicon nitrate ($SiN$) is added between the heater and the GeTe. The resulting thickness of the silicon nitrate is denoted by $h'_4$. A direct current is induced in the heater leading to a change of state in the GeTe depending on the temperature of the heater. The DC will arrive to the heater through the metal command lines. These lines are printed on a separate layer $M4'$. Furthermore, a layer of silicon dioxide of thickness ($h'_5$) is added to isolate the command lines layer $M4'$. The updated stack up and the optimized Tx ($M1'$), Rx($M3'$) in addition to the design of the command line layer ($M4'$) are reported in Fig. 9. The detailed geometrical parameters are presented in Table 3. Since the command lines are on the top layer, two stubs are integrated in the lines in order to minimize their effect on the RF.

The updated unit cell was simulated in periodic boundary conditions (PBC) with two Floquet ports under normal incidence in the same setup as in Fig. 4. The results show that the command lines increase the insertion losses; however, the phase shift $\Delta \phi$ remains 180$^{\circ }$ (Fig. 10). The minimum insertion loss of the updated cell (CL) is of 1.9 dB, whereas the minimum loss of the initial cell (NoCL) is 0.69 dB. The $-1$ dB bandwidth of the CL unit cell is 16% less (10.9%) than that of the NoCL unit cell (27%). The introduction of the command line, which paves the way for a future practical design, came at the expense of increasing the insertion losses without affecting the phase shift $\Delta \phi$.

#### Comparison of the transmitarray performances

**Figure 11:**
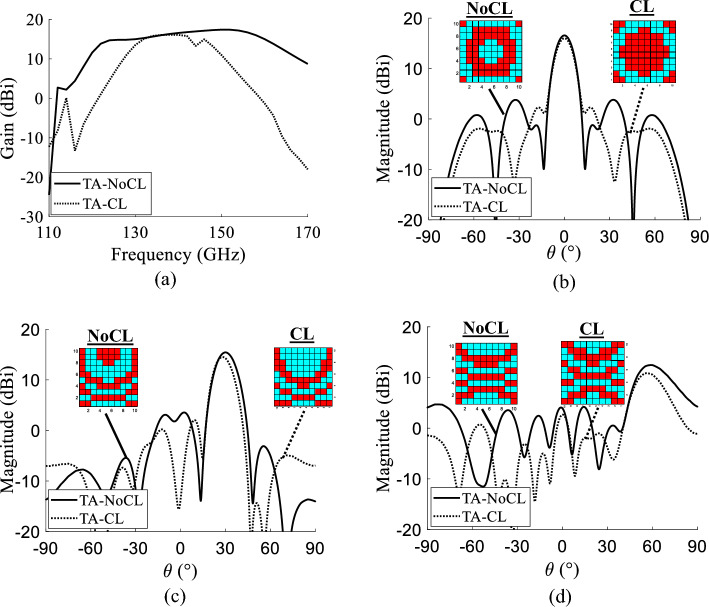
The Matlab simulation results of the transmitarray in the two cases: with command lines and without command lines.

The updated unit cell (CL) is a practical architecture of TA unit cell in the D-band, capable of steering the beam with an independent control of the GeTe. Thus, it is worthy to synthesize a TA using the MATLAB tool explained earlier in this paper and check the feasibility of such TA. A TA of $10 \times 10$ elements is considered with a focal distance of 8 mm ($F/D=$0.8), similar to the case of the TA studied at the beginning of this paper created with the initial unit cell (NoCL). The phase distributions for three transmissions are generated: (1) broadside transmission, (2) transmission towards 30$^{\circ }$ and (3) transmission towards 60$^{\circ }$. The radiation patterns of the TA constructed with the unit cell CL are compared to the radiation patterns of the TA built with the unit cell NoCL.The results show that the updated unit cell could be used in the design of TA. The TA with command lines (TA-CL) steers the beam into different directions up to 60$^{\circ }$. The comparison of the radiation patterns of the TA-NoCL and the TA-CL at 140 GHz in the case of the broadside transmission and 30$^{\circ }$ show that there is minimal difference in the main beam with slightly higher side lobes in the case of TA-CL (see Fig. 11). The difference increases in the case of the radiation pattern of the transmission towards 60$^{\circ }$. The gain versus the frequency in the case of broadside transmission is also analyzed in Fig. 11. The maximum gain at 140 GHz is almost the same (around 16.0 dBi; equivalent to an aperture efficiency (The aperture efficiency of the TA is defined as the ratio between the maximum gain and the maximum directivity of a uniform amplitude aperture with the same physical dimensions as the TA.) of 12.6%) whereas the $-1$ dB gain bandwidth is narrower in the case of TA-CL. In order to obtain a practical design, some compromises should be made such a narrower $-1$ dB gain bandwidth and higher side lobes.

## Discussion

In this paper, we introduced an electronically reconfigurable unit cell design, which is characterized by a 1-bit phase shift capability achieved by manipulating the states of two PCM-based GeTe switches. We presented an in-house Matlab tool with a hybrid approach, employed to design a TA composed of these 1-bit unit cells and conduct performance analysis. Subsequently, a full-wave simulation was executed for a 10$\times$10-element TA, focusing on patterns with 0$^{\circ }$ and 30$^{\circ }$ pointing angles, revealing the feasibility of reconfigurable TAs utilizing PCM-based switches in the D-band. To the best of our knowledge, this represents an innovative investigation into reconfigurable TAs with beam-steering capabilities in the D-band, utilizing PCM-based switches. Furthermore, we described a fabrication strategy for the entire layered structure and presented the design of command lines responsible for biasing each switch. The impact of these lines on the unit-cell performance and TA behavior is investigated. The results demonstrated that the steering capabilities remained intact, with the main lobes maintaining their original shape despite a slight increase observed in the side lobes. These compromises are made in order to achieve a novel practical design of the command lines. The study is promising for further exploration, particularly concerning fabrication processes and measurement techniques.

In conclusion, this work aims to highlight and discuss the enabling building blocks and innovative technologies (packaging, switches, bias strategy, etc.) for further promoting the application of PCM-switches and through-glass-vias solutions in sub-THz programmable electromagnetic surfaces. Note that the proposed fabrication process is not available in commercial facilities. The prototyping of a full programmable transmitarray with individually biased PCM-switches is under development in our microelectronics facilities traditionally used to manufacture CMOS integrated circuits. Several manufacturing steps must be validated before the final fabrication, e.g. management of contamination by materials which are not usually used in CMOS cleanroom facilities, adjustment of the deposition and etching process for the glass wafer having different thermal properties than the CMOS substrates, choice of the bonding technology for stack two or more glass wafer with through-glass-vias which should ensure electrical continuity between the different metal layers.

## Data Availability

The datasets used and analysed during the current study are available from the corresponding author only on reasonable request.
